# PO_2_ oscillations induce lung injury and inflammation

**DOI:** 10.1186/s13054-019-2401-1

**Published:** 2019-03-27

**Authors:** Stefan Boehme, Erik K. Hartmann, Thomas Tripp, Serge C. Thal, Matthias David, Dietmar Abraham, James E. Baumgardner, Klaus Markstaller, Klaus U. Klein

**Affiliations:** 1 Department of Anesthesia, General Intensive Care and Pain Management, Medical University of Vienna, Waehringer Guertel 18-20, 1090 Vienna, Austria; 2 Department of Anesthesiology, Medical Center of the Johannes-Gutenberg University Mainz, Mainz, Germany; 3 Department of Anesthesiology and Critical Care Medicine, KKM Catholic Medical Center Mainz, Mainz, Germany; 4 Center for Anatomy and Cell Biology, Division of Cell and Developmental Biology, Medical University of Vienna, Vienna, Austria; 5 Department of Anesthesiology, University of Pittsburgh Medical Center, Pittsburgh, PA 15261 USA

**Keywords:** Cyclic recruitment and derecruitment of atelectasis, PO_2_ oscillations, Ventilator-induced lung injury, Veno-venous extracorporeal membrane oxygenation, Pathomechanism, Lung injury, Inflammation

## Abstract

**Background:**

Mechanical ventilation can lead to ventilator-induced lung injury (VILI). In addition to the well-known mechanical forces of volutrauma, barotrauma, and atelectrauma, non-mechanical mechanisms have recently been discussed as contributing to the pathogenesis of VILI. One such mechanism is oscillations in partial pressure of oxygen (PO_2_) which originate in lung tissue in the presence of within-breath recruitment and derecruitment of alveoli. The purpose of this study was to investigate this mechanism’s possible independent effects on lung tissue and inflammation in a porcine model.

**Methods:**

To separately study the impact of PO_2_ oscillations on the lungs, an in vivo model was set up that allowed for generating mixed-venous PO_2_ oscillations by the use of veno-venous extracorporeal membrane oxygenation (vvECMO) in a state of minimal mechanical stress. While applying the identical minimal-invasive ventilator settings, 16 healthy female piglets (weight 50 ± 4 kg) were either exposed for 6 h to a constant mixed-venous hemoglobin saturation (S_mv_O_2_) of 65% (which equals a P_mv_O_2_ of 41 Torr) (control group), or an oscillating S_mv_O_2_ (intervention group) of 40–90% (which equals P_mv_O_2_ oscillations of 30–68 Torr)—while systemic normoxia in both groups was maintained. The primary endpoint of histologic lung damage was assessed by ex vivo histologic lung injury scoring (LIS), the secondary endpoint of pulmonary inflammation by qRT-PCR of lung tissue. Cytokine concentration of plasma was carried out by ELISA. A bioinformatic microarray analysis of lung samples was performed to generate hypotheses about underlying pathomechanisms.

**Results:**

The LIS showed significantly more severe damage of lung tissue after exposure to PO_2_ oscillations compared to controls (0.53 [0.51; 0.58] vs. 0.27 [0.23; 0.28]; *P* = 0.0025). Likewise, a higher expression of TNF-α (*P* = 0.0127), IL-1β (*P* = 0.0013), IL-6 (*P* = 0.0007), and iNOS (*P* = 0.0013) in lung tissue was determined after exposure to PO_2_ oscillations. Cytokines in plasma showed a similar trend between the groups, however, without significant differences. Results of the microarray analysis suggest that inflammatory (IL-6) and oxidative stress (NO/ROS) signaling pathways are involved in the pathology linked to PO_2_ oscillations.

**Conclusions:**

Artificial mixed-venous PO_2_ oscillations induced lung damage and pulmonary inflammation in healthy animals during lung protective ventilation. These findings suggest that PO_2_ oscillations represent an independent mechanism of VILI.

**Electronic supplementary material:**

The online version of this article (10.1186/s13054-019-2401-1) contains supplementary material, which is available to authorized users.

## Background

Mechanical positive pressure ventilation per se is unphysiological and can induce secondary lung injury, particularly in pre-diseased lungs [1–4]. In the last two decades, mechanistic studies addressed the harmful role of mechanical ventilation in detail [5, 6]. Biomechanical stress and strain in terms of volutrauma, barotrauma, and atelectrauma and release of inflammatory mediators (biotrauma) contribute to ventilator-induced lung injury (VILI) [7–13]. Moreover, translocation of these inflammatory mediators can induce systemic inflammation and promote a multi-organ dysfunction syndrome.

In addition to the above-mentioned mechanical mechanisms, non-mechanical pathomechanisms have been hypothesized to potentially cause or aggravate VILI [14–18]. One of these alternative mechanisms is respiration-dependent oscillations in partial pressure of oxygen (PO_2_ oscillations) which originate in lung tissue in the presence of cyclical recruitment and derecruitment of alveoli (cR/D). In different animal models of acute respiratory distress syndrome (ARDS), several studies have described the phenomenon of within-breath fluctuations in arterial PO_2_ during mechanical ventilation [19–23]. As these PO_2_ oscillations that originate in lungs by repetitive collapse and reopening of alveoli are dependent on the presence of recruitable atelectasis, they have been investigated primarily in diseased lungs. A first attempt to compare the progression of lung injury in different functional lung compartments in a surfactant depletion model combined with injurious ventilation in rabbits was presented by Otto et al., who concluded that cyclical recruitment seems more damaging than stretch injury [24].

However, the independent impact of PO_2_ oscillations on lung damage and pulmonary inflammation (biotrauma) remains unclear as their effects could not be discriminated from the other influencing biomechanical and biochemical factors prevalent during lung injury. Stimulated by studies in the field of sleep apnea, where PO_2_ alterations have been associated with higher pulmonary inflammation [25], we hypothesized that PO_2_ oscillations may represent an independent, non-mechanical contributor to VILI.

To investigate the influence of PO_2_ oscillations in a state of minimal mechanical stress, we set up an experimental in vivo animal model that allowed exposure of healthy lungs to artificially generated PO_2_ oscillations by the use of a veno-venous extracorporeal membrane oxygenation (vvECMO) system—while maintaining systemic normoxia. This model enabled us to minimize the impact of mechanical forces associated with mechanical ventilation by applying identical minimally invasive ventilator settings to both treatment groups.

In this experimental setting, we aimed to investigate in vivo if artificially induced mixed-venous PO_2_ oscillations independently cause lung damage (primary outcome parameter) and pulmonary inflammation (secondary outcome parameter) in otherwise healthy porcine lungs. Further, we wanted to learn, by bioinformatic microarray analysis of lung samples, which pathways might be involved in the pathology linked to PO_2_ oscillations.

## Methods

### Animal experiments

After the State Animal Care Committee approval (Landesuntersuchungsamt Koblenz, Rhineland Palatinate, Germany: 23 177-07/G 10-1-047), 20 healthy female piglets (50 ± 4 kg) were studied. Four pilot experiments were necessary to set up the vvECMO model. The remaining *N* = 16 animals were randomly assigned to either the control or the intervention group. Two of the animals assigned to the control group expired during ECMO cannulation. Thus, *N* = 6 subjects of the control group and *N* = 8 subjects of the intervention group were included to the final analysis.

### Anesthetic procedures and routine monitoring

All experimental procedures were performed after induction and maintenance of general anesthesia by continuous infusion of propofol and fentanyl. Besides standard monitoring, a left ventricular catheter, two ECMO cannulas (right internal jugular vein and right femoral vein), a left-sided femoral arterial introducer to advance an indwelling PO_2_ sensing probe to the ascending aorta, and a central venous introducer for Swan-Ganz catheterization were placed by the Seldinger technique under ultrasound guidance in sterile manner. Hemodynamic and respiratory parameters were recorded using a Datex-Ohmeda-Monitor system (Datex-Ohmeda, Inc., Madison, USA) and stored via the iCollect software (BORQ Pte Ltd., Singapore). The animals remained in dorsal recumbency for the entire experiment.

### Continuous mixed-venous oxygen hemoglobin saturation measurement

Mixed-venous oxygen hemoglobin saturation (S_mv_O_2_) was assessed by a Swan-Ganz catheter positioned in the pulmonary artery. S_mv_O_2_ was measured based on the principle of reflection spectrometry at a sampling frequency of 1 Hz. Data was read out by the Edwards Vigileo monitor (Edwards Lifesciences, Irvine, USA). The monitor device was calibrated before each measurement time point against a mixed-venous blood gas analysis, so as to compensate for the conditions of the hemoglobin disassociation curve. Intermittent cardiac output (CO) was assessed by a single indicator transpulmonary thermodilution of three repetitive bolus injections of 10 mL cold normal saline.

### Real-time measurement of arterial partial pressure of oxygen

To assess the arterial partial pressure of oxygen (P_a_O_2_) in real-time, we used a calibrated ruthenium-tipped oxygen-sensing probe (Foxy AL300, OceanOptics, Dunedin, USA) that was advanced to the ascending aorta. The PO_2_ probe was connected via a fiberglass cable to a server unit (NeoFox, OceanOptics, Dunedin, USA). This PO_2_ measurement based on the principle of fluorescence quenching of oxygen by the multi-frequency phase fluorimetry (MFPF) method has been previously published [26]. PO_2_ values were digitally sampled with a temporal resolution of 10 Hz and stored on a personal computer.

### Veno-venous extracorporeal membrane oxygenation

After insertion of the ECMO cannulas (Sorin Group GmbH, Munich, Germany) into the right femoral vein (23/25 Fr.) and right internal jugular vein (23 Fr.), they were connected to a prefilled ECMO system with heparin-coated 3/8 in. silicone tubing. The ECMO system consisted of a multi-flow roller pump (S3, Stöckert, Munich, Germany), a heat exchanger (System 3 T, Stöckert, Munich, Germany), an oxygenator (Jostra Quadrox D, Maquet Cardiopulmonary AG, Hirrlingen, Germany), and an arterial filter. After cannulation, anticoagulation was induced with an intravenous heparin bolus (100 IU/kg), followed by a continuous infusion targeting an activated clotting time of 180–220 s.

**Fig. 1:**
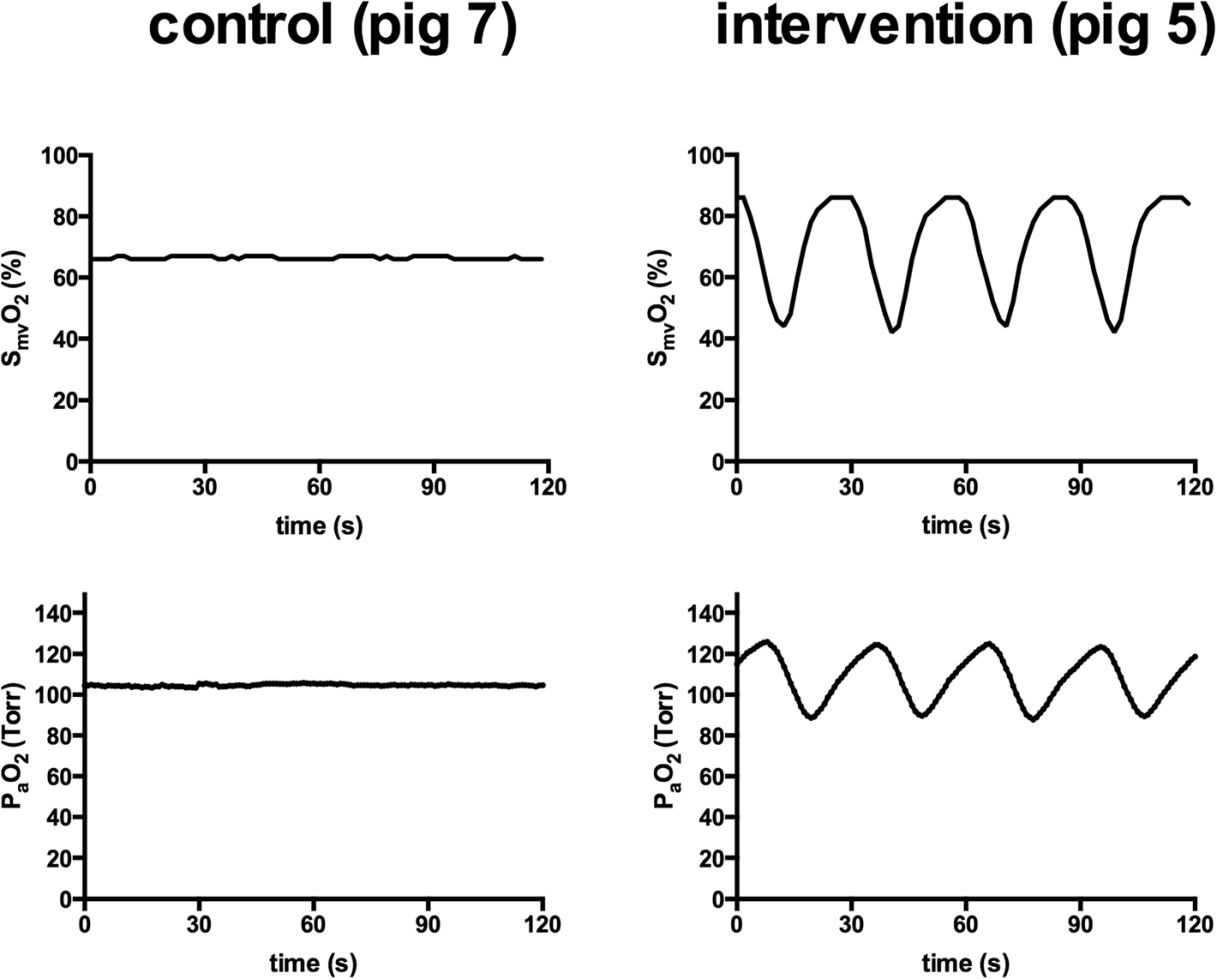
Conditions of oxygenation. Mixed-venous hemoglobin oxygen saturation (S_mv_O_2_) and the resulting arterial partial pressure of oxygen (P_a_O_2_). The left side shows the measures in one of the control animals (constant PO_2_), on the right side the measures in one of the intervention animals (artificially induced PO_2_ oscillations)

To control the gas concentrations and flow rates of oxygen (O_2_), medical air (AIR), and nitrogen (N_2_) over the oxygenator, we used a custom-built computer-controlled O_2_/AIR/N_2_-switch that allowed the application of any O_2_ concentration and flow pattern. In particular, we could program a specific flow pattern (e.g., apply an endless loop of 6 L/min O_2_ for 15 s, followed by 4.8 L/min N_2_ for 7 s, followed by zero flow for 8 s to generate reproducible artificial PO_2_ oscillations) and adjust the settings online. Thus, we were able to generate both, constant partial pressures of oxygen and artificial PO_2_ oscillations via the vvECMO system. An example of the resulting conditions is given in Fig. 1.

### Study protocol

Prior to vvECMO implementation, a baseline healthy measurement (BLH) of hemodynamic parameters, respiratory parameters, arterial and mixed-venous blood gas analyses were assessed together with Swan-Ganz (S_mv_O_2_) and MFPF (P_a_O_2_) recordings during standardized ventilation settings: tidal volume (*V*_T_) of 6 mL/kg body weight, positive end-expiratory pressure (PEEP) of 5 cm H_2_O, inspiration to expiration (I:E) ratio of 1:2, inspiratory fraction of oxygen (F_I_O_2_) of 0.21–0.3 and variable respiratory rate (RR) to obtain a partial pressure of carbon dioxide (P_a_CO_2_) of 35–45 Torr. According to our protocol, we defined a P_a_O_2_/F_I_O_2_ ratio < 450 as dropout criterion.

For both the control and intervention group, the vvECMO was implemented and blood flow of 3.5–4 L/min was set by adjustment of the rpm speed. The sweep gas flow over the oxygenator was titrated to permissive hypercapnia, defined as P_a_CO_2_ of 40 to 60 Torr. Further, we defined a target mean P_a_O_2_ of 100 Torr, as measured by the reference method of real-time P_a_O_2_ sensing. The fraction of delivered O_2_ applied via the oxygenator of the vvECMO system was titrated with the help of the computer-controlled O_2_/AIR/N_2_ switch. This regimen allowed for the identical, minimally invasive ventilator settings in both the intervention and the control group that were defined as follows: continuous positive airway pressure (CPAP) of 15 cm H_2_O and ultra-low intermittent positive pressure ventilation with a fixed *V*_T_ of 2 mL/kg body weight, RR of 20 breath/min, and I:E ratio of 1:1 at F_I_O_2_ of 0.3–0.4.

To maintain hemodynamic stability throughout the entire experiment, we kept mean arterial pressure (MAP) above 60 mmHg. Besides a continuous infusion of Sterofundin (B Braun, Melsungen, Germany) of 4 mL/kg/h, the animals received additional boluses of 50 mL hydroxyl ethyl starch (HAES 6%) if MAP was below 60 mmHg, until the response of the fluid bolus on systolic arterial pressure was less than a 5 mmHg. Thereafter, the animals could receive norepinephrine in steps of 0.1 μg/kg/min.

Then, after a 30 min stabilization period of vvECMO implementation, the animals were either exposed for 6 h to a constant S_mv_O_2_ of 65% (control group) or an oscillating S_mv_O_2_ of 40–90% with a cycle frequency of 2 per minute (intervention group)—as artificially induced by variation in fractional O_2_ delivery and sweep gas flow via the computer-controlled O_2_/AIR/N_2_ switch, while maintaining systemic normoxia (mean P_a_O_2_ 100 Torr). Measurements were taken 30 min after vvECMO initiation (BL-ECMO) and after 6 h (ECMO 6) in analogy to BLH. Additionally, at time point BL-ECMO and ECMO 6, EDTA blood samples were withdrawn from the left ventricular catheter for enzyme-linked immunosorbent assay (ELISA) and for blood count analysis. After the defined 6 h of experimental time, the animals were euthanized with an overdose of fentanyl and propofol and the lungs were exsanguinated and then removed. The left lung was fixed at a CPAP level of 15 cm H_2_O in 4% buffered formalin and stored for subsequent histologic analysis. From the right lung, duplicate tissue samples from apical, middle and basal lung areas were collected, shock-frozen, and stored at − 80 °C for quantitative real-time polymerase chain reaction (qRT-PCR) and microarray analysis.

### Assessment of histologic lung damage

The formalin-fixated lung tissue blocks from apical, central and basal lung region were paraffined, microtome-sliced, and hematoxylin and eosin (H&E) stained. Of each lung region (tissue block), 5 representative photomicrographs were carried out at 60-fold image magnification (210 photomicrographs in total) and analyzed in blinded fashion by two observers independently (TT and DA). Here, we used a previously reported lung injury score (LIS) by Matute-Bello et al. [27] for semi-quantitative assessment of lung damage by judging the following parameters: (i) neutrophils in alveolar space, (ii) neutrophils in interstitial space, (iii) hyaline membranes, (iv) protein detritus in the alveolar space, and (v) septum thickening. Scoring was performed according to the previously presented thresholds [27], and the weighted LIS was calculated as follows: [20 × (i) + 14 × (ii) + 7 × (iii) + 7 × (iv) + 2 × (v)]/100—resulting in values from 0 to 1, where 1 represents the maximal possible lung damage.

### Assessment of pulmonary inflammatory response

As a measure of pulmonary inflammation, we quantified the mRNA copies from the lung tissue samples by qRT-PCR for the pro-inflammatory cytokines of interleukin (IL)-1β (IL-1β), IL-6, and tumor necrosis factor (TNF-α) and of the enzymes inducible nitric oxide synthase (iNOS) and prostaglandin G/H synthase-2 (PGH2)—using a Lightcycler™ 480 PCR system (Roche Applied Science, Rotkreuz, Switzerland). All measures were normalized versus the control gen *Sus scrofa* peptidyl isomerase A (PPIA), as described in previous studies [28, 29]. The used primers are given in the supplement (Additional file 1: Table S1).

### Assessment of systemic inflammatory response

From the centrifuged EDTA plasma samples, the concentrations of the cytokines TNF-α, IL-1β, and IL-6 were carried out quantitatively by commercially available ELISA kits (Porcine TNF-alpha Quantikine ELISA Kit, Porcine IL-1 beta/IL-1F2 Quantikine ELISA Kit, Porcine IL-6 Quantikine ELISA Kit, R&D Systems Europe, Ltd., Abingdon, UK) with lower detection limits of 5 pg/mL for TNF-α and IL-6 and of 13.6 pg/mL for IL-1β.

### Gene expression analysis for hypothesis generation

From the lung tissue samples (mixed samples from apical, central, and basal) of randomly chosen two control and two intervention animals, microarray analysis was performed for hypothesis generation. RNA target preparation was performed using the GeneChip 3′ IVT Express Kit (Affymetrix). End-labeled cDNAs were applied to GeneChip Porcine Genome Arrays (Affymetrix) and scanned using GeneChip® Scanner 3000 and Affymetrix GeneChip Command Console Software (AGCC). For the analysis of microarray data, the empirical Bayes method was applied by using the statistical software R (v 3.1.1) with the limma package (v 3.20.9) [30, 31].

#### Ingenuity Pathway Analysis

Data were analyzed through the use of QIAGEN’s Ingenuity® Pathway Analysis (IPA®, QIAGEN Redwood City, http://www.qiagen.com/ingenuity). By comparing the imported microarray data generated with Ingenuity® Knowledge Base, a list of relevant canonical pathways was obtained.

Predictions of the activation status of pathways were done by using IPA Upstream Regulator Analysis Tool by calculating a regulation *z*-score and an overlap *P* value, which were based on the number of known target genes of pathways and expression changes of these target genes. The pathways were generated through the use of Ingenuity Target Explorer (QIAGEN, https://targetexplorer.ingenuity.com).

### Statistical analysis

The primary outcome parameter of lung damage (LIS) was addressed by the Wilcoxon-signed rank test. Wilcoxon signed-rank test was also performed for the secondary outcome parameter of pulmonary inflammation and for the side observation of systemic inflammation. Due to the exploratory character of this study, no correction for multiplicity was performed. The bioinformatics microarray analysis (for hypothesis generation) was carried out using the empirical Bayes method. Data are presented as median with 25% and 75% quartile (Q1; Q3) or by the mean and standard deviation (SD). Additionally, descriptive statistics have been performed to outline potential group differences in the hemodynamic, oxygenation, and clinical chemistry measures by a generalized linear model analysis for repeated measures (with group as between-subject factor and ECMO—i.e., different time points—as within-subject factor)—as data was normally distributed (Kolmogorov-Smirnov test passed). Statistics were performed using the statistical software GraphPad Prism v6 (GraphPad Software Inc., San Diego, CA, USA).

## Results

At BLH, all animals appeared to be lung healthy (with a Horowitz index of > 500), exhibiting normal values in dynamic respiratory system compliance for the controls (mean, 1.25 ± 0.21 mL/cm H_2_O/kg), as well as for the intervention group (mean, 1.24 ± 0.2 mL/cm H_2_O/kg). None of the subjects had to be excluded from the study. Routinely, the study protocol predefined inclusion criteria could be achieved in all animals. In the controls, a constant mean S_mv_O_2_ of 66 ± 2.8% with less than 3% of fluctuation was achieved (equivalent to a mixed-venous PO_2_ from 40.1 to 43.3 Torr) that resulted in a constant mean P_a_O_2_ of 104.3 ± 3.7 Torr, with less than 5 Torr of fluctuation. In contrast, in the intervention group, oscillations in S_mv_O_2_ around a mean of 67.5 ± 2.8% with an amplitude of 41.1 ± 5.1% were induced by variations of fractional O_2_ delivery and sweep gas flow over the oxygenator of the vvECMO. The corresponding nadir and peak values for mixed-venous PO_2_ were 31.9 and 65.1 Torr. The resulting average P_a_O_2_ was 103.1 ± 5 Torr with a mean oscillation amplitude (Δ P_a_O_2_) of 29.4 ± 7.2 Torr. For hemodynamic support, each animal received 8 to 13 boluses of 50 mL HAES 6% throughout the experiment, and none of the animals required norepinephrine. The applied respiratory parameters, vvECMO settings, hemodynamic variables, results of blood gas analysis and P_a_O_2_ sensing, and clinical chemistry (blood count) data are summarized in Table 1.

**Table 1:** Respiration, hemodynamic, and oxygenation parameters

Measurement time point	Control (constant PO_2_)			Intervention (cyclic PO_2_)		
	BLH	BL-ECMO	ECMO 6	BLH	BL-ECMO	ECMO 6
Respiration						
RR (per min)	22 ± 4	20	20	23 ± 3	20	20
*V*_T_ (mL/kg)	6.2 ± 0.4	2	2	6.1 ± 0.3	2	2
PEEP/CPAP (cm H_2_O)	5 ± 1	15	15	5 ± 1	15	15
I to E ratio	1:1	1:1	1:1	1:1	1:1	1:1
F_I_O_2_	0.3	0.4	0.4	0.3	0.4	0.4
vv-ECMO						
ECMO-blood flow (L/min)	NA	3.6 ± 0.2	3.7 ± 0.4	NA	3.5 ± 0.1	3.8 ± 0.5
Sweep gas flow (L/min)	NA	4.4 ± 0.8	4.8 ± 0.8	NA	3.9 ± 0.9	4 ± 0.6
FO_2_ oxygenator	NA	60.7 ± 13.1	56.5 ± 7.9	NA	1–100	1–100
Hemodynamics						
HR (per min)	95 ± 13	111 ± 6	103 ± 6	99 ± 11	100 ± 12	103 ± 16
MAP (mm Hg)	80.5 ± 9.6	84.3 ± 8.3	79.1 ± 11.7	83.2 ± 8.3	75 ± 9.9	79.3 ± 7.5
MPAP (mm Hg)	21.2 ± 1.6	22.9 ± 2.7	21.8 ± 3	20.4 ± 2.4	22.5 ± 3.4	23 ± 3.8
CVP (mm Hg)	12 ± 2	12 ± 2	12 ± 3	12 ± 2	13 ± 1	11 ± 2
CO (L/min)	5.5 ± 1.2	5.8 ± 0.6	5.1 ± 0.9	5.2 ± 0.5	5.1 ± 1	4.6 ± 0.6
Oxygenation						
P_a_CO_2_ (Torr)	40.2 ± 6.4	51.9 ± 11.1	55.8 ± 3.9	38.8 ± 4.6	47.8 ± 6.9	52.1 ± 4.1
Mean P_a_O_2_ (Torr)	175.3 ± 19.6	105.3 ± 4.8	102.6 ± 2.8	162.7 ± 22.8	106.2 ± 7.8	102 ± 2.6
ΔP_a_O_2_ (Torr)*	4.1 ± 1.8	3.5 ± 1.6	2.3 ± 1.3	4.4 ± 2.4	30.5 ± 6.8	27 ± 9.4
Mean S_mv_O_2_ (%)	69.4 ± 6.6	66.3 ± 2.8	66 ± 3.4	66.2 ± 7.1	67.4 ± 3	69 ± 2.7
ΔS_mv_O_2_ (%) *	3 ± 1.6	2.4 ± 1.1	1.6 ± 0.9	3 ± 0.8	38.5 ± 7.2	40.9 ± 4.3
P_mv_O_2_ (Torr)	44.7 ± 5.5	44.9 ± 2.5	42.1 ± 2.3	42.9 ± 6	42.6 ± 5.4	48.1 ± 6.2
S_p_O_2_ (%)	99 ± 1	99 ± 1	99 ± 1	99 ± 1	99 ± 1	99 ± 1
Clinical chemistry						
Hemoglobin (g/dL)	NA	8.9 ± 0.9	7.3 ± 1	NA	9.0 ± 0.7	7.8 ± 0.5
Red blood cells (T/L)	NA	5.9 ± 0.8	4.5 ± 0.4	NA	5.5 ± 0.3	4.8 ± 0.3
White blood cells (G/L)	NA	24 ± 4.7	20.3 ± 5.4	NA	26.5 ± 7.2	23.1 ± 0.3
Platelets (G/L)	NA	347 ± 89	264 ± 72	NA	330 ± 173	233 ± 135

Presented are the results of respiration and vvECMO settings, hemodynamics and oxygenation parameters and clinical chemistry (blood count) data. Values are given as mean and standard deviation, itemized for the control group (constant PO_2_) and the intervention group (cyclic PO_2_) for the measurement time points of baseline healthy (BLH), after vvECMO installation (BL-ECMO), and after 6 h of the experimental time (ECMO 6). Descriptive statistics to point out differences between the groups: generalized linear model analysis for repeated measures

*RR* respiratory rate, *V*_T_ tidal volume, *PEEP* positive end-expiratory pressure, *CPAP* continuous positive airway pressure, *I to E ratio* inspiration to expiration ratio, *F*_*I*_*O*_*2*_ fraction of inspired oxygen, *vv-ECMO* vevo-venous extracorporeal membrane oxygenation, *HR* heart rate, *MAP* mean arterial pressure, *MPAP* mean pulmonary artery pressure, *CVP* central venous pressure, *CO* cardiac output, *P*_*a*_*CO*_*2*_ arterial partial pressure of carbon dioxide, *P*_*a*_*O*_*2*_ arterial partial pressure of oxygen, *ΔP*_*a*_*O*_*2*_ oscillation amplitude in P_a_O_2_, *S*_*mv*_*O*_*2*_ mixed venous oxygen saturation, *ΔS*_*mv*_*O*_*2*_ oscillation amplitude in S_mv_O_2_, *P*_*mv*_*O*_*2*_ mixed venous partial pressure of oxygen, *S*_*p*_*O*_*2*_ peripheral capillary oxygen saturation

*P < 0.001

### Histologic lung damage

**Fig. 2:**
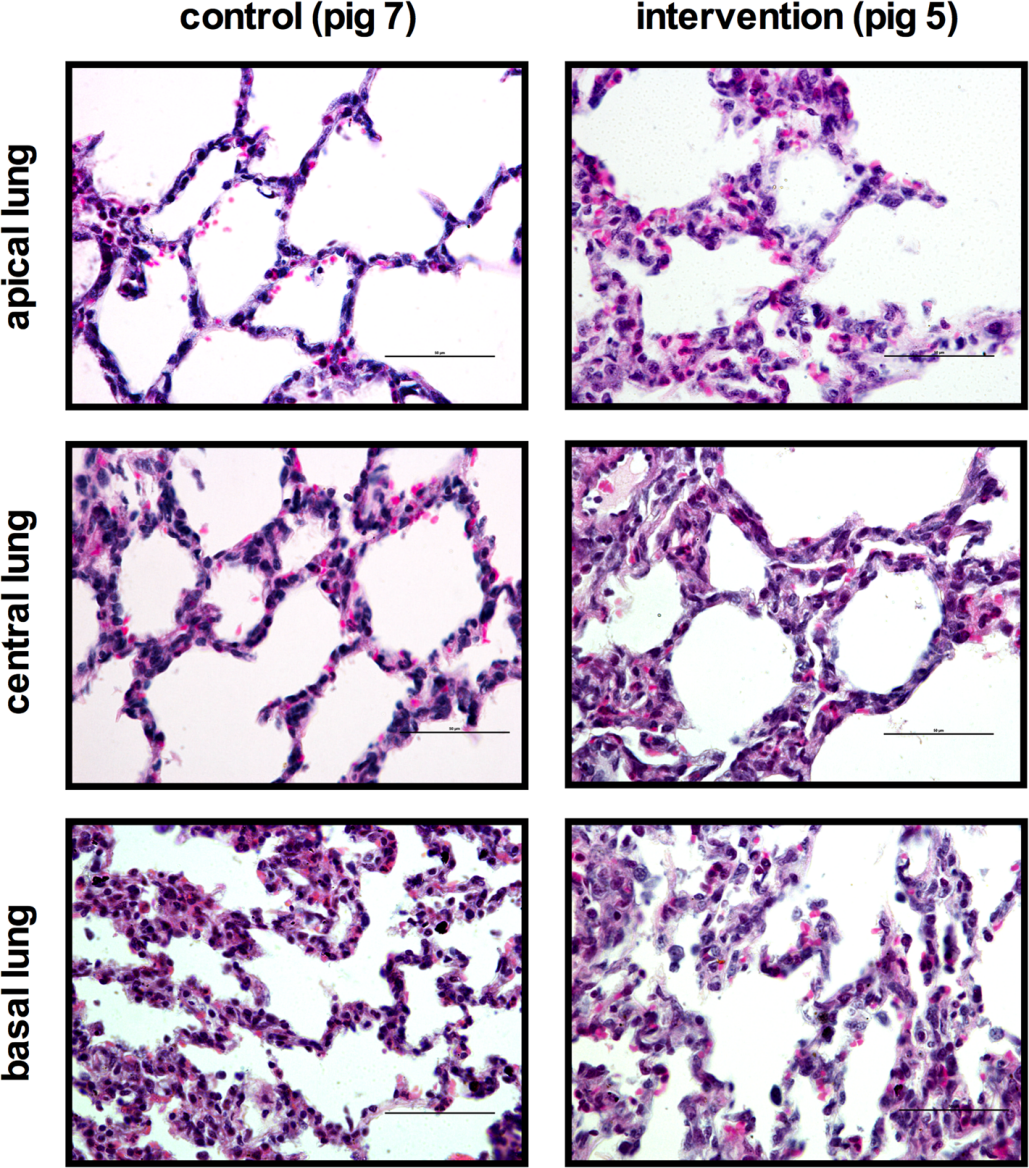
Representative photomicrographs from the lungs stained with H&E. On the left side images of the control group and on the right of the interventions group are presented for the investigated lung region of interest (allocation apical, central, and basal lung). Histologic images are given in 60-fold magnification with the scale presenting 50 μm

Control group animals showed healthy alveolar structures, with sporadic neutrophils. In contrast, intervention group animals showed pronounced infiltration of inflammatory cells and morphologic changes of lung tissue. Inflammatory changes included, predominantly, a largely extended number of neutrophils in alveolar and interstitial space after the exposure of PO_2_ oscillations with slightly more protein detritus in the alveolar space (Fig. 2). Only minor structural changes, i.e., of alveolar remodeling with light alveolar septal thickening and hyaline membranes, were observed.

These findings were also reflected by the post-processed LIS. In comparison to controls, the LIS showed significantly higher values in the intervention group for the entire lung 0.53 (0.51; 0.58) vs. 0.27 (0.23; 0.28); *P* = 0.0025). This lung damage was also evident in all subregions examined—with highest LIS values found in the dependent lung regions. The detailed results with regard to the lung regions examined are presented in Table 2. The factors of the weighted LIS that had the largest influence were neutrophil infiltration in alveolar and in interstitial space as well as protein detritus in the alveolar space, as highlighted in Table 3.

**Table 2:** Results of “lung injury scoring”

Lung injury score (LIS)—median (Q1; Q3)			
	Control	Intervention	*P* value
Entire lung	0.27 (0.23; 0.28)	0.53 (0.51; 0.58)	0.0025
Apical lung	0.24 (0.16; 0.27)	0.52 (0.32; 0.60)	0.0023
Central lung	0.24 (0.15; 0.39)	0.50 (0.29; 0.59)	0.0047
Basal lung	0.33 (0.18; 0.46)	0.59 (0.46; 0.70)	0.001

Lung injury was assessed correspondingly to Matute Bello et al.—using a semi-quantitative weighted lung injury scoring (LIS) that contained the following parameters: (i) neutrophils in alveolar space, (ii) neutrophils in interstitial space, (iii) hyaline membranes, (iv) protein detritus in alveolar space, and (v) septum thickening. LIS was calculated as follows: [20 × (i) + 14 × (ii) + 7 × (iii) + 7 × (iv) + 2 × (v)]/100—resulting in values from 0 to 1, where 1 represents maximal lung damage. Results are presented for the different lung regions of interest (allocation) investigated and summarized for the entire lung. Measures are given as median and 25% and 75% quartile (Q1; Q3). *P* value: Wilcoxon signed-rank test

**Table 3:** Results of “lung injury scoring” parameters

Lung injury score (LIS) parameters—median (Q1; Q3)			
	Control	Intervention	*P* value
(i) Neutrophils in alveolar space	0.04 (0.01; 0.08)	0.2 (0.18; 0.24)	0.0013
(ii) Neutrophils in interstitial space	0.16 (0.13; 0.16)	0.22 (0.21; 0.23)	0.0025
(iii) Hyaline membranes	0.008 (0; 0.04)	0.05 (0.01; 0.06)	0.1768
(iv) Protein detrius in alveolar space	0.008 (0; 0.016)	0.04 (0.02; 0.05)	0.0025
(v) Septum thickening	0.01 (0.01; 0.01)	0.02 (0.02; 0.02)	0.0303

Lung injury scoring (LIS) itemized for the following judged factors of (i) neutrophils in alveolar space, (ii) neutrophils in interstitial space, (iii) hyaline membranes, (iv) protein detritus in alveolar space, and (v) septum thickening. Score per field was assessed semi quantitatively: (i) was scored 0 if none, 1 if 1 to 5, and 2 if higher 5; (ii) was scored 0 if none, 1 if 1 to 10, and 2 if higher 10; (iii) was scored 0 if none, 1 if 1, and 2 if higher 1; (iv) was scored 0 if none, 1 if 1, and 2 if higher 1; (v) was scored 0 if none, 1 if 2 to 4, and 2 if 5 or higher. For LIS calculation, the factors (i) to (v) were weighted by the following formula: [20 × (i) + 14 × (ii) + 7 × (iii) + 7 × (iv) + 2 × (v)]/100. Results are presented as median with 25% and 75% percentile (Q1; Q3). *P* value: Wilcoxon signed-rank test

### Pulmonary inflammatory response

**Fig. 3:**
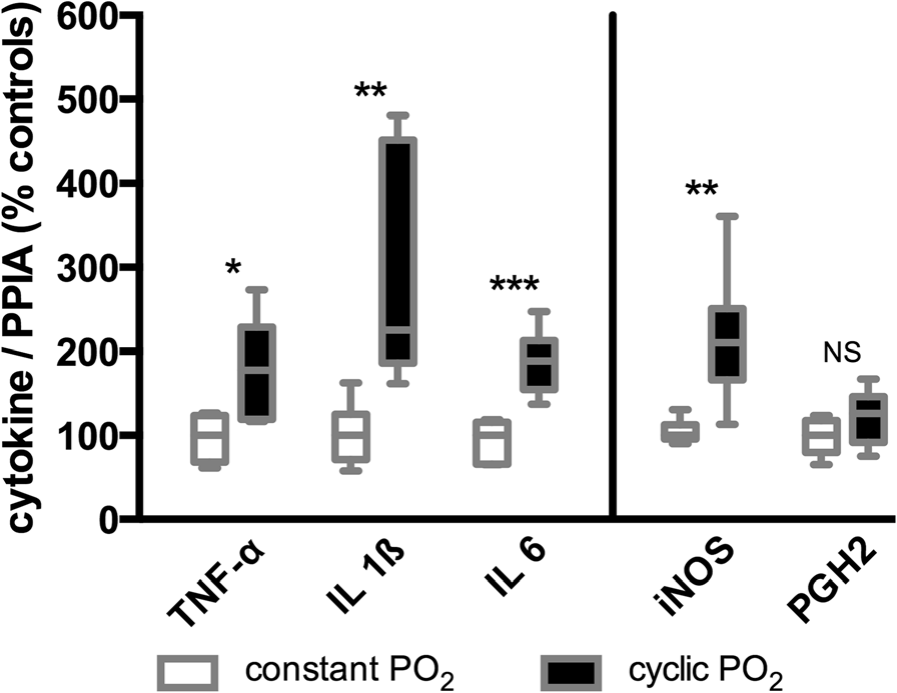
Results of the investigated genes in lung tissue by quantitative real-time polymerase chain reaction. The plot shows the percentage differences in cytokine copy numbers versus *Sus scrofa* peptidylprolyl isomerase A (PPIA) between the control group (constant PO_2_) and the intervention group (cyclic PO_2_). TNF-α, *Sus scrofa* tumor necrosis factor; IL-1β, *Sus scrofa* interleukin 1 beta; IL-6, *Sus scrofa* interleukin 6; iNOS, *Sus scrofa* nitric oxide synthase 2; PGH2, *Sus scrofa* prostaglandin G/H synthase-2. Statistical significance was carried out by the Wilcoxon signed-rank test: NS ≥ 0.05; 0.01 ≤ * < 0.05; 0.001 ≤ ** < 0.01; 0.001 ≤ *** < 0.0001. Box plots represent the median, 25% and 75% percentile, whiskers the minimum and maximum values

In order to evaluate gene expression levels of potential inflammatory effectors in the PO_2_ oscillation group, we performed qRT-PCR on a selected panel of inflammatory genes. mRNA expression of TNF-α, IL-6, IL-1β, and iNOS differed between the groups. In the intervention group, we found significantly higher values for TNF-α (*P* = 0.0127), IL-1β (*P* = 0.0013), IL-6 (*P* = 0.0007), and iNOS (*P* = 0.0013) as compared to the control animals (Fig. 3). Concerning PGH2, slightly but not significantly higher values could be detected (*P* = 0.2231). The absolute copy numbers normalized versus PPIA are presented in the supplement (Additional file 1: Table S2).

### Systemic inflammatory response

**Fig. 4:**
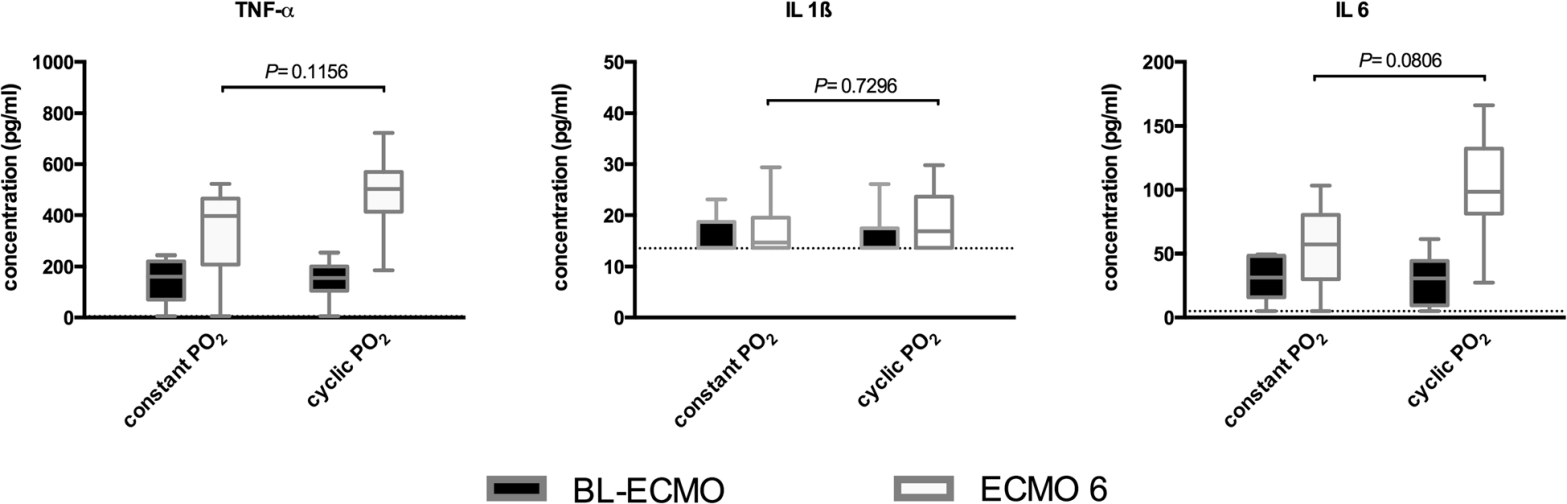
ELISA results in plasma. Showing the plasma concentrations of TNF-α, IL-6 and IL-1β after initiation of vvECMO (BL-ECMO) and after 6 h of experimental time (ECMO 6) for either constant PO_2_ or PO_2_ oscillations. Box plots represent the median, 25% and 75% percentile, whiskers the minimum and maximum values. The dashed line the lower detection limits. *P* values calculated by Wilcoxon signed-rank test

ELISA analyses of plasma samples showed almost identical concentrations for TNF-α, IL-6, and IL-1β at BL-ECMO in both study groups. Cytokine concentrations increased in both groups after 6 h (ECMO 6); however, differences between the control group (constant PO_2_) and the intervention group (cyclic PO_2_) reached no statistical significance (Fig. 4).

### Ingenuity Pathway Analysis of transcriptome changes induced by PO_2_ oscillations—hypothesis generation

To identify the underlying molecular factors that might play a role in the pathology associated with PO_2_ oscillations—in terms of hypothesis generation—a porcine microarray analysis was performed. PO_2_ oscillations led to significant changes in gene expression as identified by the top 100 genes with the highest change in gene expression. For these genes, the adjusted *P* value was *P* < 0.0001 and log2 fold change (log2FC) ranged from − 6.4 to 2.46. The heat map for these 100 genes is available in the supplement (Additional file 2: Figure S1). Of those 100 genes, 57 could be attributed to human analogs (Additional file 2: Table S3).

In order to search for pathways involved in PO_2_ oscillations, we used the Ingenuity Pathway Analysis (IPA) to functionally annotate the obtained differentially expressed genes and to investigate the potential biological relevance of the transcriptional changes. To this end, the dataset was categorized using IPA Canonical Pathways. Relevant canonical pathways that turned out from this analysis included pathways which are involved in inflammation, namely glucocorticoid receptor signaling (*P* = 5.34E−23; overlap 39.3%), acute phase response signaling (*P* = 7.11E−17; overlap 30.5%), FXR/RXR activation (*P* = 2.62E−14; overlap 37%), and LXR/RXR activation (*P* = 7.52E−14; overlap 37.2%).

Pathway analysis underlined transcriptome changes after exposure of PO_2_ oscillations and predicted changes in pathway activation. Analysis of up and downregulated genes after PO_2_ oscillation was also performed with respect to canonical pathways using IPA. Calculation of an activation *z*-score was used to infer the activation states of pathways.

**Fig. 5:**
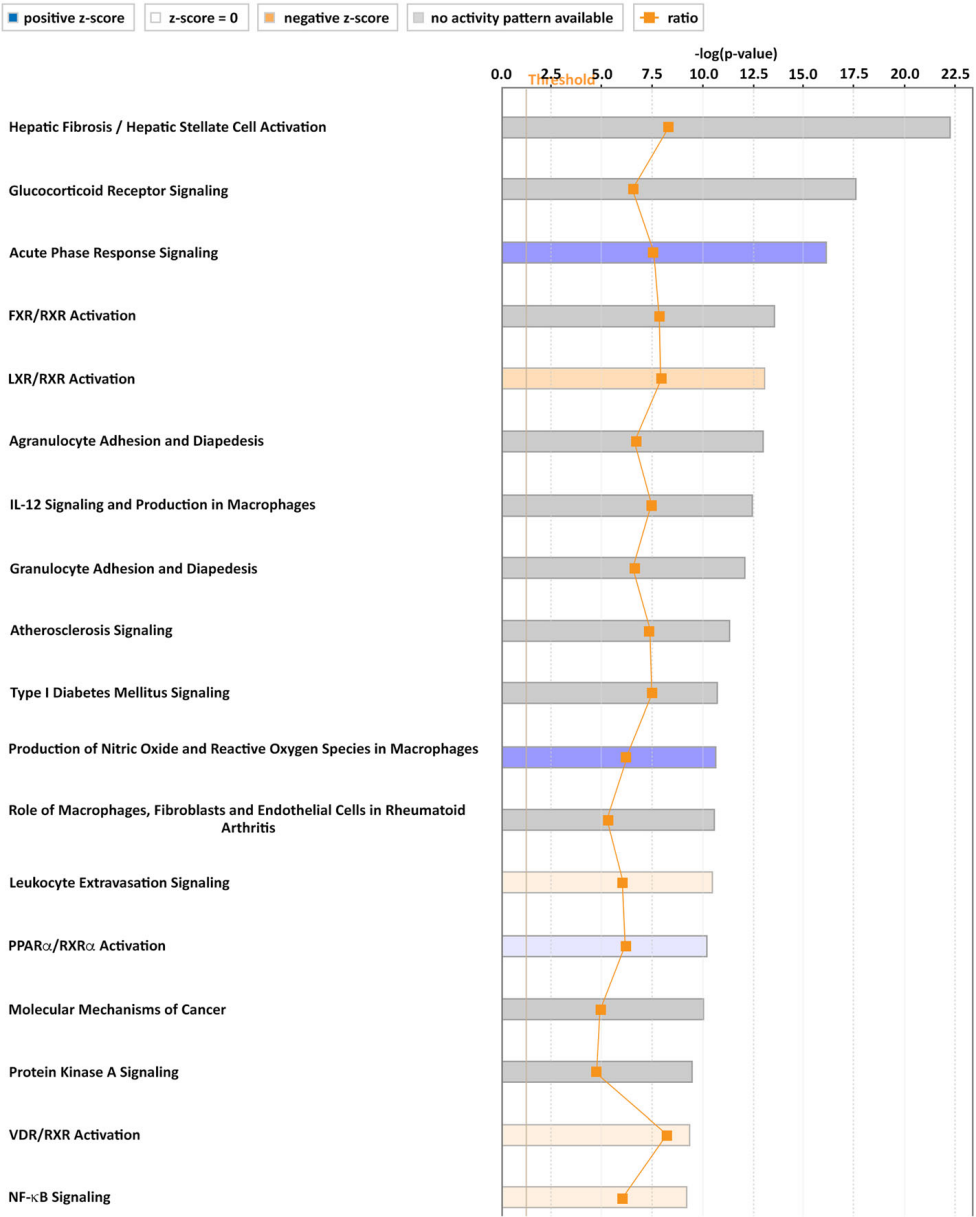
Top regulated canonical pathways based on their *z*-score analyzed through Ingenuity Pathway Analysis. PO_2_ oscillations resulted in significantly altered levels of mRNAs enriched in canonical pathways predicting a changed activation status of several pathways. The calculated *z*-score indicates a pathway with genes exhibiting overall increased mRNA levels (blue bars) or decreased mRNA levels (orange bars). The ratio (orange dots connected by a line) indicates the ratio of genes from the dataset that map to the pathway divided by the total number of genes that map to the same pathway

PO_2_ oscillations resulted in significantly altered levels of mRNAs that were enriched in canonical pathways thereby predicting activated or inhibited pathways such as acute phase response signaling, LXR/RXR activation, production of nitric oxide and reactive oxygen species in macrophages, leukocyte extravasation signaling, PPARa/RXRa activation, VDR/RXR activation, and NF-kB signaling, all of them related to inflammation (Fig. 5).

Based on the abovementioned canonical pathways, predicted changed activation status of pathways and our qRT-PCR data, we analyzed selected inflammation-related pathways generated through the use of Ingenuity Target Explorer (QIAGEN) in our microarray data set.

We analyzed interactions among the deregulated genes associated with signal-transduction pathways of IL-1, IL-6, TNF-α, interferon γ (INFγ), and LXR/RXR activation and provide evidence that the pathways of IL-6 signaling (32 deregulated genes detected: 18 up and 14 downregulated) and production of nitric oxide and reactive oxygen species (NO−/ROS) in macrophages (53 deregulated genes detected: 32 up and 21 downregulated) seem to be affected by the observed changes in gene expression. The detailed results are provided in the supplement (Additional file 2: Figure S2-S4).

## Discussion

The present study investigated the effects of PO_2_ oscillations on lung damage and pulmonary inflammation in a state of minimal mechanical stress. The study design used a vvECMO system that exposed healthy lungs to artificial mixed-venous PO_2_ oscillations during ventilation with ultra-protective settings. Results show that PO_2_ oscillations induced pulmonary neutrophil infiltration and morphologic lung damage. Likewise, inflammation-related and differentially expressed genes in lung tissue were documented after the exposure of PO_2_ oscillations, while systemic cytokine levels did not differ significantly between the groups. The bioinformatics microarray analysis of lung tissue predicted changes in inflammatory pathway activation and suggests that IL-6 and NO−/ROS signaling pathways might play a role in the pathology associated with PO_2_ oscillations.

The main challenge to address our study hypothesis was to develop a model that allowed discriminating the potential impact of PO_2_ oscillations from the known biomechanical forces (i.e., volutrauma, barotrauma, and atelectrauma) and biochemical mechanisms (i.e., biotrauma) induced by injurious mechanical ventilation. We implemented an experimental setup in pigs that artificially induced PO_2_ oscillations to otherwise healthy lungs by the use of a vvECMO system in a setting of minimal stress and strain caused by mechanical ventilation. The chosen vvECMO settings enabled us to ventilate with an ultra-low tidal volume of 2 mL/kg bodyweight, while keeping the lungs open with a CPAP of 15 cm H_2_O, which has been proven lung protective [32]. By computer-controlled adjustment of O_2_ delivery and sweep gas flow through the oxygenator of the vvECMO system, we were able to set a continuous S_mv_O_2_ of 66% (control group) or artificially induce PO_2_ oscillations around a mean S_mv_O_2_ of 67%, ranging from 47% to 88% (intervention group). Real-time P_a_O_2_ sensing documented the maintenance of systemic normoxia (at least 80 Torr of P_a_O_2_) in both the control and the intervention group. The S_mv_O_2_-oscillations were targeted for a range that could be reproducibly achieved with ECMO sweep gas changes and accurately tracked by the continuous oximetry catheter. The corresponding mixed-venous PO_2_ oscillations were 31.9 to 65.1 Torr. Oscillations in lung tissue PO_2_ and local pulmonary capillary PO_2_ during cyclical recruitment and derecruitment of alveoli have never been measured, but potentially could be substantially larger than our induced mixed-venous PO_2_ oscillations. During the collapse, with no gas space and no gas exchange, lung tissue and pulmonary capillary PO_2_ should approach mixed-venous PO_2_. After recruitment, with a gas space composed only of water vapor, CO_2_, and O_2_ (after denitrogenation, if F_I_O_2_ = 1.0), lung tissue PO_2_ and pulmonary capillary PO_2_ could approach a very high PO_2_. PO_2_ oscillations with an amplitude of several hundred Torr have been measured in a systemic artery after surfactant depletion and during mechanical ventilation with settings expected to promote cyclical recruitment [21, 23]. Thus, compared to the clinical scenario where portions of the lung could be cyclically recruited, with large PO_2_ swings, for many hours, our induced PO_2_ oscillations were fairly small and of short duration.

To address the primary outcome parameter of lung injury caused by the exposure of PO_2_ oscillations we chose a well-established semi-quantitative LIS by Matute Bello et al. [27]. This histological assessment of lung injury has been proposed to capture the pathological hallmarks of early acute lung injury (ALI), as defined by diffuse alveolar damage. Histologically, diffuse alveolar damage is characterized by neutrophilic alveolitis; fibrin-rich proteinaceous exudates in the airspaces; deposition of hyaline membranes; interstitial thickening; and microvascular thrombi (i.e., endothelial injury and activation of the coagulation cascade) [33, 34]. These multiple histological features of ALI were mainly covered by the post-processed LIS parameters. Our results showed a significantly higher extent of lung injury—as assessed by LIS—after exposure of artificial PO_2_ oscillation, which was predominantly caused by neutrophil accumulation and deposition of proteinaceous debris. Due to the fact that we studied otherwise healthy lungs, the LIS scores in the recent study were moderate compared to classical ARDS models. Araos and colleagues, e.g., found values of 0.8 to almost 1 (by use of the identical LIS) in a porcine severe ARDS vvECMO model; however, their sham-operated group revealed almost the identical LIS values that we found in our vvECMO control group [35]. The observed LIS findings after cyclic PO_2_ exposure in the present study seem reasonable as they reflect the predominant characteristic feature of tissue injury typically reported in the context of VILI - namely alveolar infiltration of neutrophils [27] accompanied by local inflammation within the lungs. In the present study, the intrapulmonary inflammatory response was evaluated by qRT-PCR. Compared to the controls, we found significantly higher gene expression of TNF-α, IL-1β, IL-6, and iNOS in lung tissue - indicating increased synthesis in the early stages of pulmonary inflammation after exposure to PO_2_ oscillations. Although ECMO therapy is associated with several adverse effects [36, 37] that include activation of the coagulation and complement system, leukocyte activation and cytokine release, in our study both the control and treatment groups were identically exposed to ECMO.

In terms of hypothesis generation, we additionally conducted a microarray analysis. We have performed a comparative gene expression analysis of PO_2_ oscillations versus controls and have identified significant transcriptome changes after exposure of PO_2_ oscillations. Of the top 100 effected genes, 57 were related to human analogs. IPA analysis of the differentially expressed genes led to the identification of canonical pathways involved in inflammation. Together with our qRT-PCR data, we identified the signal transduction pathways of IL-1, IL-6, TNF-α, and interferon gamma (IFNγ)—attributable to the pathways of IL-6 signaling, production of NO and ROS, and the acute phase response signaling that may represent changed inflammatory activity in lungs with PO_2_ oscillations. These signaling pathways are involved in chemotaxis and adhesion of neutrophils, and superoxide and ROS production, which might partly explain the observed histological pathology linked to PO_2_ oscillations. The LXR/RXR signaling network may contribute to regulation of the expression level of inflammatory mediators, including IL-6, IL-1, and iNOS. The identified pathways, however, are involved in the regulation of several other signal transduction pathways, which might explain why we found not only upregulated but also downregulated gene expression changes. Further, the observed changes in gene expression of the involved factors do not necessarily reflect changes in the activity of the mentioned signaling pathways—as activation and deactivation of signaling pathways are regulated through post-translational modifications such as receptor binding or phosphorylation.

### Limitations

One methodological limitation of our approach was that the maximal frequency of artificially induced PO_2_ oscillations was only 2 per minute, while alveolar tissue PO_2_ oscillations might occur at the rate of the respiratory frequency.

We also cannot totally exclude the possibility that our minimally invasive ventilation regimen and the vvECMO per se might have contributed to the observed lung damage – although both were performed in an identical manner in both groups.

Concerning the microarray analysis, the lung tissue samples contained multiple cell types without isolation of specific cell types. Therefore, changes in pathways that are primarily associated with specific cell types, for example, NO production by macrophages, cannot in our analysis be specifically attributed to those cell types.

Finally, we used the vvECMO as an experimental tool for creating PO_2_ oscillations, and our study offers no insight into the clinical management of ECMO.

## Conclusions

As demonstrated experimentally by the vvECMO model, artificially induced mixed-venous PO_2_ oscillations were associated with acute lung damage and pulmonary inflammation in healthy animals during lung protective ventilation. As a first hypothesis, IL-6 related signaling pathways—including acute phase response signaling—and NO−/ROS pathways appear to be involved in the pathology caused by PO_2_ oscillations. These findings suggest that PO_2_ oscillations may represent an alternative mechanism in the development of VILI.

## Additional files

Additional file 1: Containing the supplementary **Table S1.** and **Table S2.** (PDF 83 kb)

Additional file 2: Containing the supplementary **Figures S1-S4.** and **Table S3.** (PDF 1590 kb)

## Abbreviations

ARDS: Acute respiratory distress syndrome
BL: Baseline
CO: Cardiac output
CPAP: Continuous positive airway pressure
cR/D: Cyclical recruitment and derecruitment of alveoli
CVP: Central venous pressure
ELISA: Enzyme-linked immunosorbent assay
F_I_O_2_: Fraction of inspired oxygen
H&E: Hematoxylin and eosin
HR: Heart rate
I:E: Inspiration to expiration ratio
IL-1β: Interleukin 1β
IL-6: Interleukin 6
iNOS: Inducible nitric oxide synthase
LIS: Histologic lung injury scoring
MAP: Mean arterial pressure
MPAP: Mean pulmonary artery pressure
mRNA: Messenger ribonucleic acid
P_a_CO_2_: Arterial partial pressure of carbon dioxide
P_a_O_2_: Arterial partial pressure of oxygen
PEEP: Positive end-expiratory pressure
PGH2: Prostaglandin G/H synthase-2
P_mv_O_2_: Mixed venous partial pressure of oxygen
PO_2_: Partial pressure of oxygen
PPIA: Peptidyl isomerase A
qRT-PCR: Quantitative real-time polymerase chain reaction
RR: Respiratory rate
S_mv_O_2_: Mixed-venous hemoglobin saturation
S_p_O_2_: Peripheral capillary oxygen saturation
TNF-α: Tumor necrosis factor alpha
VILI: Ventilator-induced lung injury
*V*_T_: Tidal volume
vvECMO: Veno-venous extracorporeal membrane oxygenation
ΔP_a_O_2_: Oscillation amplitude in P_a_O_2_
ΔS_mv_O_2_: Oscillation amplitude in S_mv_O_2_
