# Supplementary material for: PO2 oscillations induce lung injury and inflammation
Source: Crit Care. 2019 Mar 27;23:102. doi: 10.1186/s13054-019-2401-1 (PMC6438034; doi:10.1186/s13054-019-2401-1)
Supplement: Supplementary file 1 — Containing the supplementary Table S1. and Table S2. (PDF 83 kb) [file 13054_2019_2401_MOESM1_ESM.pdf]

Additional File 1

Specific probes und primers used for qRT-PCR

PCR Probe	Oligonukleotidsequence (5'-3')	GenBank Nr.
**iNOS**	S: 5'-gATggCACCATCATAggggAC-3'<br>A: 5'-ggCACCCTgggAACTCAA-3'<br>FL: 5'-TGGAACACCCCAAATACGAGTGGTTCC-FL<br>CY5: L670-GGAGCTGGAGCTGAAGTGGTACGCCC-Phos	NM_001143690
**TNF-α**	S: 5'-CCCAgAAggAAgAgTTTCCA-3'<br>A: 5'-CggCTTTgACATTggCTACA-3'<br>FL: 5'-ggCCCAAggACTCAgATCATCgTC-FL<br>CY5: L670-CAAACCTCAgATAAgCCCgTCgC-Phos	NM_214022
**IL-1β**	S: 5'-ACCCTgCAgCTggAggAT-3'<br>A: Red-CCTTTggAgTTTCCCAggA-3'	NM_214321
**IL-6**	S: 5'-CCAATCTgggTTCAATCAggA-3'<br>A: 5'-gTggTggCTTTgTCTggATTC-3'<br>FL: 5'-TgTCgAggCTgTgCAgATTAgTACCA-FL<br>CY5: L670-gCACTgATCCAgACCCTgAggCAA-Phos	NM_214399
**PGH2**	S: 5'-CCCCTTCTgCCTgACgC-3'<br>A: 5'-CTCTgCTCTggTCgATTgAgg-3'<br>FL: 5'-TCTATCTTACTggAACATggCATCACCCA-FL<br>CY5: L670-TTTgTTgAATCATTTAgCAggCAAATTgCT-Phos	NM_214321
**PPIA**	S: 5'-CTTTCACAgAATAATTCCAggATT-3'<br>A: 5'-ggACAAgATgCCAggACC-3'<br>FL: 5'-ATgCTTCAggATAAAATTCTCATCATCAAA-FL<br>CY5: L670-TTCTCTCCATAgATggACTTgCCACCA-Phos	NM_214353

Table S1: Probes and Primers used for quantitative real-time polymerase chain reaction (qRT-PCR). S: sense Primer; A: anti-sense Primer; FL: Fluoreszin; CY5: CY5-coupled, Phos: Phosphate; iNOS: Sus scrofa nitric oxide synthase 2, inducible (iNOS), mRNA; TNF-α: Sus scrofa tumor necrosis factor (TNF superfamily, member 2) (TNF-α), mRNA; IL-1β: Sus scrofa interleukin 1 beta (IL-1β), mRNA; IL-6: Sus scrofa interleukin 6 (interferon, beta 2) (IL-6), mRNA; PGH2: Sus scrofa prostaglandin G/H synthase-2 (PGH2), mRNA; PPIA: Sus scrofa peptidylprolyl isomerase A (PPIA), mRNA.

Absolute copy numbers of investigated genes – median (Q1; Q3)		
	**Control group (Copy numbers / PPIA)**	**Intervention group (Copy numbers / PPIA)**
**TNF-α**	0,0217 (0,0164; 0,0258)	0,0385 (0,0264; 0,0434)
**IL-1ß**	0,00232 (0,00184; 0,00260)	0,00523 (0,00441; 0,00998)
**IL-6**	0,294 (0,215; 0,328)	0,554 (0,457; 0,596)
**iNOS**	0,000742 (0,000726; 0,000780)	0,002667 (0,001493; 0,001785)
**PGH2**	0,943 (0,801; 1,087)	1,191 (0,985; 1,354)

Table S2: Absolute copy numbers of investigated genes by quantitative real-time polymerase chain reaction (qRT-PCR) from lung tissue. TNF-α: Sus scrofa tumor necrosis factor; IL-1β: Sus scrofa interleukin 1 beta; IL-6: Sus scrofa interleukin 6; iNOS: Sus scrofa nitric oxide synthase 2; PGH2: Sus scrofa prostaglandin G/H synthase-2. Results are presented as median, 25% and 75% quartile (Q1; Q3).
