# Supplementary material for: PO2 oscillations induce lung injury and inflammation
Source: Crit Care. 2019 Mar 27;23:102. doi: 10.1186/s13054-019-2401-1 (PMC6438034; doi:10.1186/s13054-019-2401-1)
Supplement: Supplementary file 2 — Containing the supplementary Figures S1-S4. and Table S3. (PDF 1590 kb) [file 13054_2019_2401_MOESM2_ESM.pdf]

Additional File 2

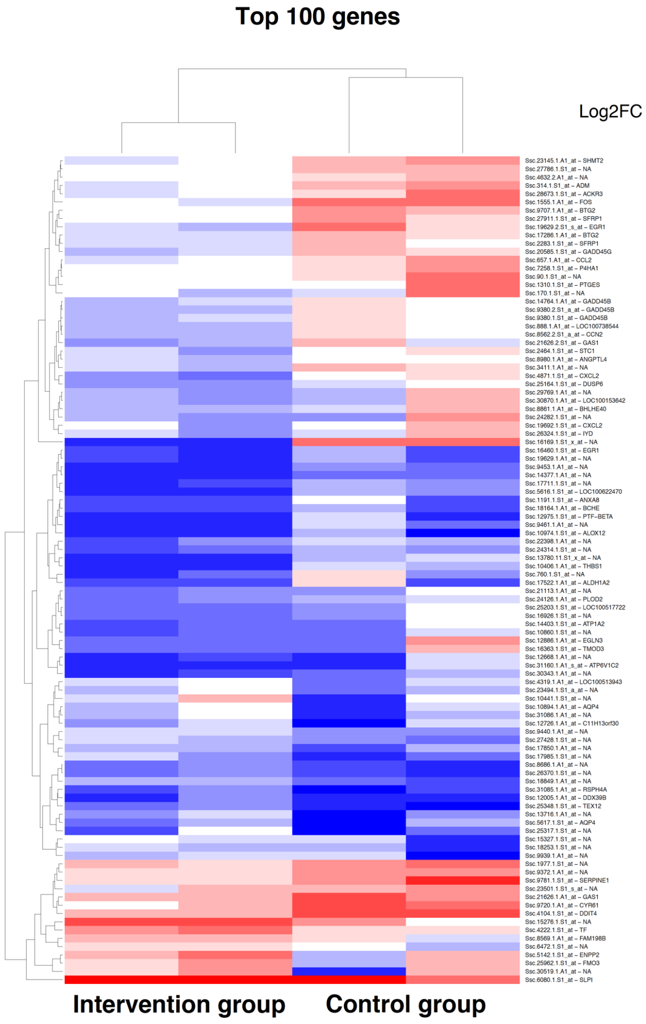

Figure S1. Heat-Map of the Top 100 genes. Heat-Map showing different expression patterns of the top 100 genes with adjusted P-value $p<0.0001$ and log2 fold change (log2FC) ranging from 1.23 to 6.4 in up-regulated genes and from -2.46 to -1.25 in down-regulated genes in the intervention group compared with control level gene expression. The heat map indicates up-regulated (blue) and down-regulated (red) gene expression. The columns represent individual tissue samples including two intervention samples and two control samples. The rows are labeled with individual gene symbols. NA indicates not attributable to human genome database.

Table S3. Top 100 differentially expressed genes showing the highest values of fold change for the intervention group.

Gene	log2FC	adjusted P-value
SLA-DRB1	6.41	6.18E-97
SLA-2	2.97	2.32E-19
FOS	2.94	4.17E-19
---	2.72	3.49E-16
LOC100518542	2.57	2.41E-14
SERPINE1	2.47	3.98E-13
LOC100154721	-2.46	4.03E-13
C17H20orf85	-2.39	2.58E-12
CXCL2	2.39	2.58E-12
---	2.34	8.25E-12
CYR61	2.28	3.48E-11
CES1 /// CES3 /// LOC100517530	2.27	4.11E-11
LOC100514063	-2.26	5.33E-11
---	2.18	3.03E-10
---	2.12	1.26E-09
ADM	2.10	1.99E-09
TMOD3	2.01	1.53E-08
---	-1.98	2.73E-08
LOC100515346	1.94	6.59E-08
LOC100516459	1.91	1.22E-07
LOC100153821	1.91	1.26E-07
---	-1.87	3.04E-07
---	1.84	4.98E-07
BTG2	1.84	5.32E-07
---	-1.76	2.39E-06
LOC396828	1.75	2.95E-06
GADD45G	1.75	2.95E-06
---	-1.72	5.06E-06
---	-1.70	6.36E-06
ARG1	1.69	7.45E-06
---	1.69	8.50E-06
ZFP36	1.68	1.00E-05
---	1.67	1.07E-05
IYD	1.65	1.52E-05
LOC100525528	1.64	1.76E-05
---	1.63	2.01E-05
ENPP2	-1.63	2.27E-05
CXCL2	1.62	2.33E-05
---	-1.61	3.06E-05
LOC100520981	1.59	3.83E-05
---	1.59	4.19E-05
---	1.58	4.27E-05
BTG2	1.58	4.61E-05
---	1.58	4.64E-05
LOC100155930	1.57	5.07E-05
FABP4	1.56	6.41E-05
PTF-BETA	1.54	8.93E-05
LOC100153642	1.52	1.29E-04

---	-1.51	1.29E-04
---	1.51	1.29E-04
LOC100514873	1.51	1.30E-04
---	1.50	1.44E-04
CCL2	1.50	1.60E-04
---	-1.47	2.33E-04
---	1.47	2.68E-04
---	1.46	2.68E-04
LOC100514063	-1.44	3.98E-04
---	1.43	4.48E-04
CCN2	1.43	4.59E-04
---	1.42	4.82E-04
---	-1.41	5.63E-04
ATP6V1C2	1.41	6.44E-04
---	-1.40	6.47E-04
---	1.39	7.44E-04
SPOCK3	-1.39	7.78E-04
---	-1.39	7.78E-04
---	1.39	7.78E-04
LOC100523562	-1.38	9.06E-04
TF	-1.37	9.78E-04
---	1.37	1.04E-03
PLOD2	1.36	1.14E-03
STC1	1.36	1.15E-03
LOC100516459	1.36	1.18E-03
PTGES	1.36	1.19E-03
---	-1.35	1.29E-03
LOC100157364	-1.34	1.56E-03
---	1.33	1.61E-03
SLPI	-1.33	1.82E-03
P4HA1	1.31	2.14E-03
PTPRC	-1.31	2.30E-03
---	-1.30	2.49E-03
---	-1.30	2.73E-03
---	1.29	2.78E-03
LOC100518542	1.29	2.78E-03
---	1.29	2.81E-03
ALOX12	1.29	3.07E-03
---	-1.28	3.34E-03
---	1.28	3.48E-03
CHI3L1	1.27	3.82E-03
LOC100156294	1.27	3.84E-03
---	1.27	3.84E-03
LOC100513943	-1.26	4.06E-03
---	1.26	4.07E-03
SNAPC3	-1.26	4.25E-03
---	-1.25	4.40E-03
LOC100522314	1.25	4.47E-03
ANGPTL4	1.25	4.47E-03
---	-1.25	4.47E-03
---	1.24	5.31E-03
---	1.23	5.64E-03

acute phase response signaling

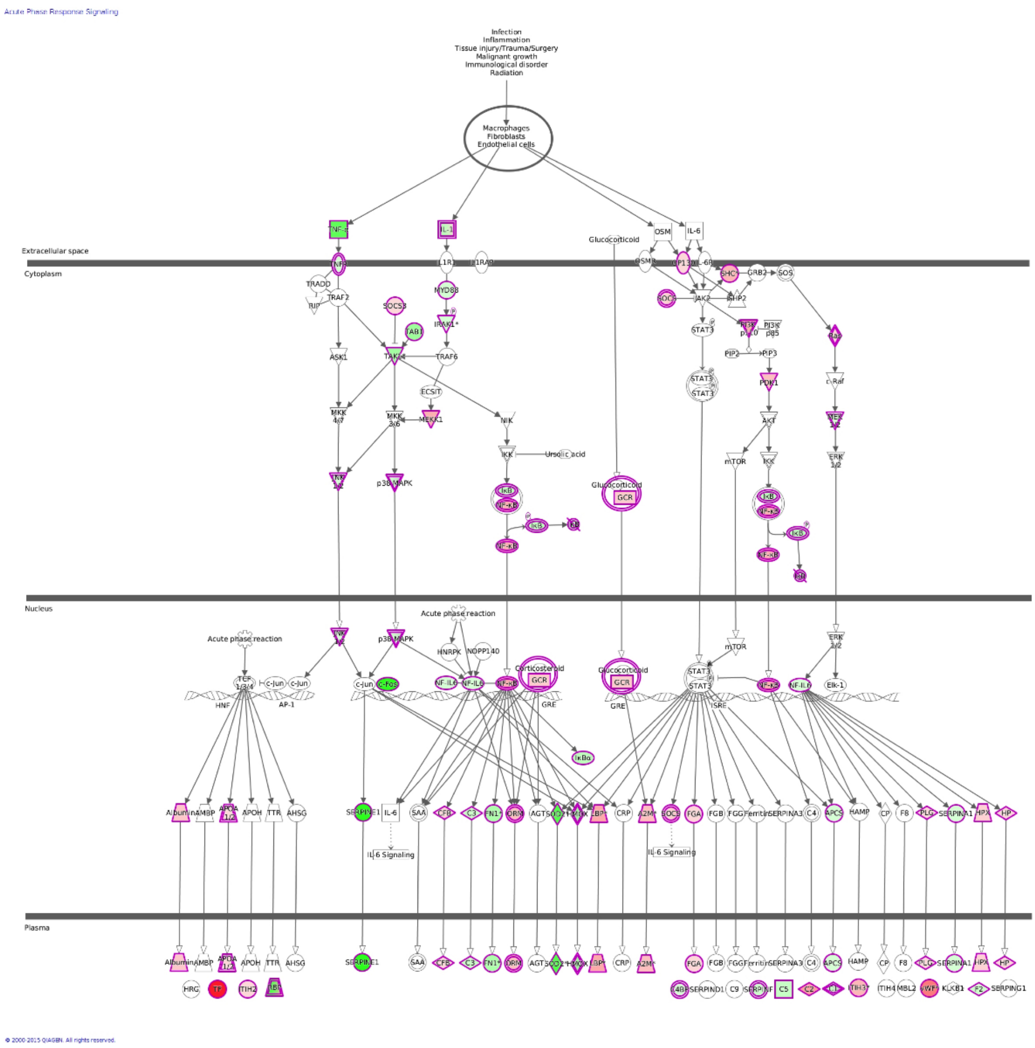

Figure S2. Acute phase response signaling network involving deregulated genes in lungs exposed to PO_2-oscillations – identified using INGENUITY pathway analysis. Up-regulated genes are shown in green and down-regulated genes are shown in red. Data presented are the results of the microarray analysis of two control and two intervention animals with the human genome database as reference.

LXR/RXR signaling network

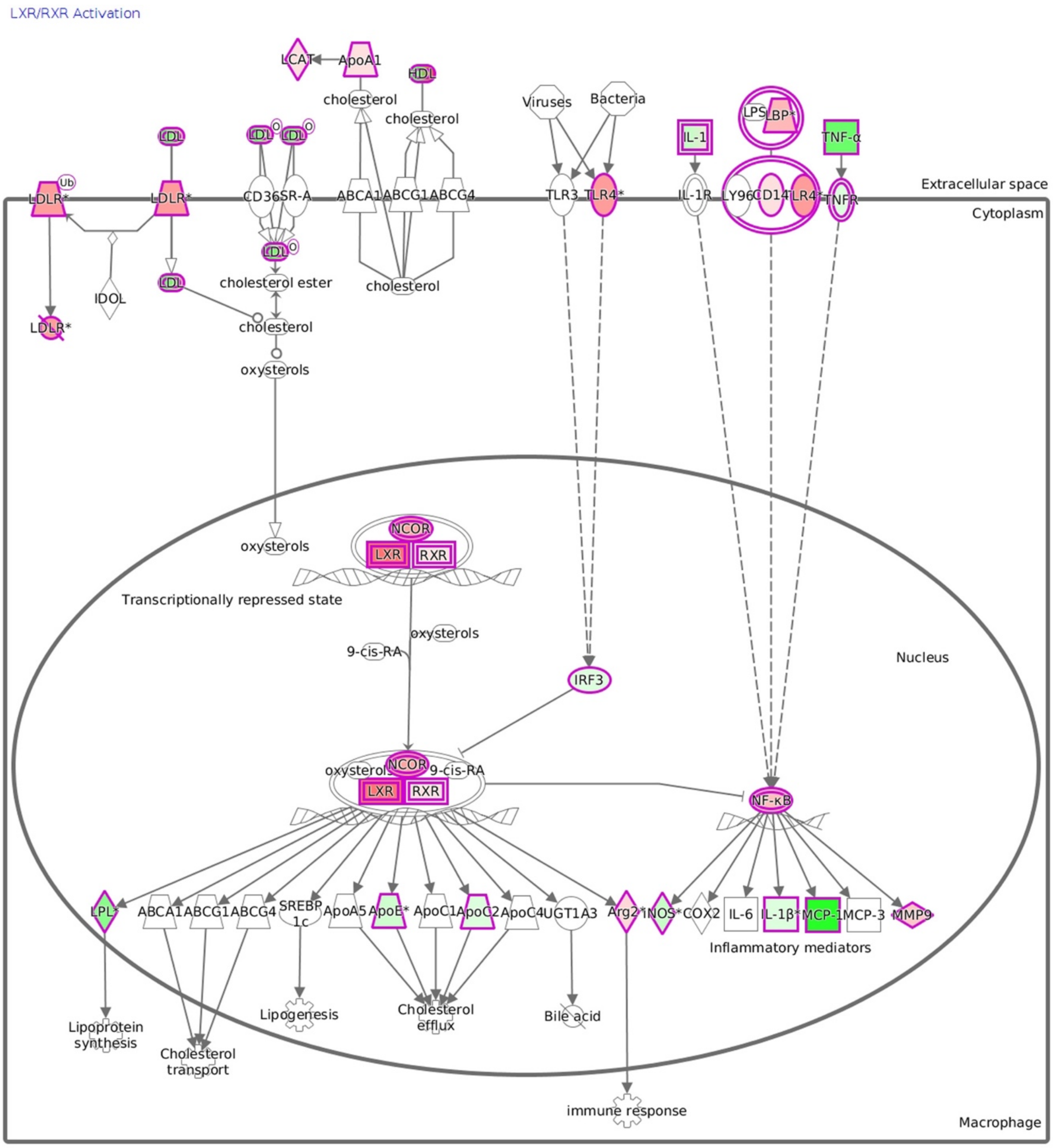

Figure S3. LXR/RXR signaling network involving deregulated genes in lungs exposed to PO_2-oscillations – identified using INGENUITY pathway analysis. Up-regulated genes are shown in green and down-regulated genes are shown in red. Data presented are the results of the microarray analysis of two control and two intervention animals with the human genome database as reference.

IL 6 signaling

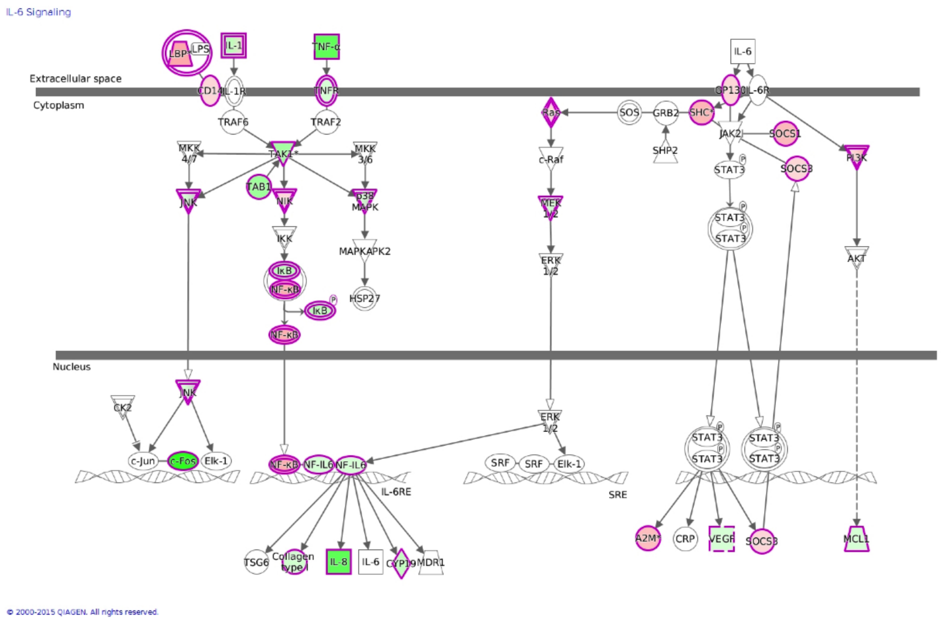

NO/ROS signaling

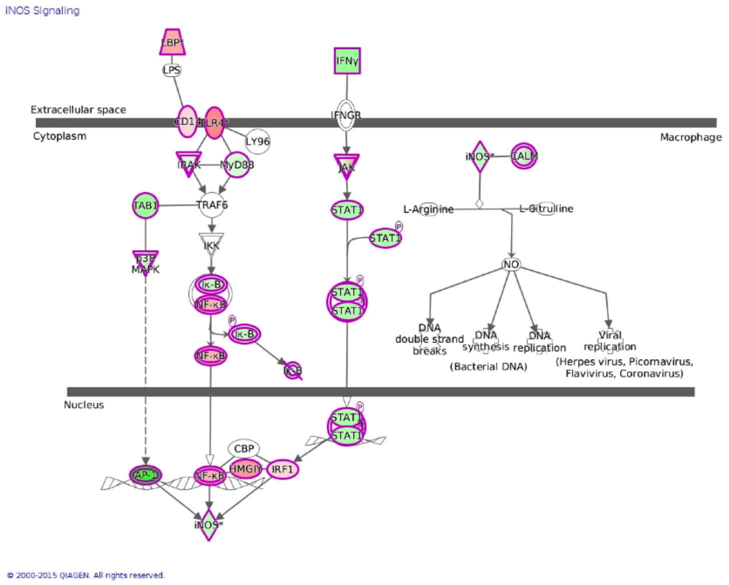

Figure S4. IL-6 and NO/ROS-signaling networks involving deregulated genes in lungs exposed to PO_2-oscillation – identified using INGENUITY pathway analysis. Up-regulated genes are shown in green and down-regulated genes are shown in red. Data presented are the results of the microarray analysis of two control and two intervention animals with the human genome database as reference.
